# Biocide Use in the Antimicrobial Era: A Review

**DOI:** 10.3390/molecules26082276

**Published:** 2021-04-14

**Authors:** Imogen Anne Jones, Lovleen Tina Joshi

**Affiliations:** School of Biomedical Sciences, University of Plymouth, Plymouth PL4 8AA, UK; imogen.jones@students.plymouth.ac.uk

**Keywords:** biocides, bacteria, antibiotic, antimicrobial, disinfection, surfaces, transmission, chemical, resistance

## Abstract

Biocides are widely used in healthcare and industry to control infections and microbial contamination. Ineffectual disinfection of surfaces and inappropriate use of biocides can result in the survival of microorganisms such as bacteria and viruses on inanimate surfaces, often contributing to the transmission of infectious agents. Biocidal disinfectants employ varying modes of action to kill microorganisms, ranging from oxidization to solubilizing lipids. This review considers the main biocides used within healthcare and industry environments and highlights their modes of action, efficacy and relevance to disinfection of pathogenic bacteria. This information is vital for rational use and development of biocides in an era where microorganisms are becoming resistant to chemical antimicrobial agents.

## 1. Introduction

Biocides are antimicrobial chemical agents that are used heavily within domestic, industry and healthcare environments for disinfection purposes [1]. The use of biocides, such as chlorinated handwash used by 19th Century physician Ignaz Semmelweis, have become integral over the centuries in the control of infections and in individual patients alongside the use of antibiotics [1,2,3]. Today, biocides comprise disinfectants and topical agents such as antiseptics and preservatives including, but not limited to, quaternary ammonium compounds (QACs), biguanides, chlorine-releasing agents and peroxygens [1,4,5]. Scientific advancement has allowed biocidal chemicals to be applied across various items, such as surgical scrubs, mouthwashes, soaps and socks, to prevent infection [6].

However, the increased use of biocides at different ranges of concentrations has led to significant scientific debate regarding their role in bacterial survival and resistance [5,7]. Indeed, studies have revealed bacterial resistance to biocides, such as chlorine resistance in *Salmonella typhi*, which has given credence to the argument that ineffectual biocide use can cause selective pressure in bacteria, which subsequently respond to develop resistance mechanisms [7,8,9]. Similarly, bacteria have developed methods of antibiotic resistance in response to the overuse of antibiotics. Thus, combined, bacterial resistance to antibiotics and biocides presents a significant challenge to address if we are to tackle antimicrobial resistant infections appropriately [9]. In an era where infection control is seen as a key method of preventing transmission of antimicrobial resistant microorganisms, biocide effectiveness must be retained. This review provides a summary of common biocides used in disinfection of bacteria, and scientific evidence of the emergence of bacterial resistance against critical biocides.

## 2. Quaternary Ammonium Compounds (QACs)

**Figure 1:**
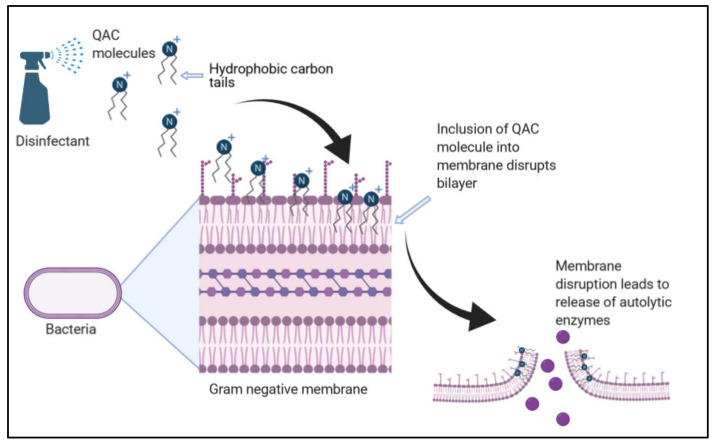
The bactericidal process by quaternary ammonium compound (QAC) disinfectants. The hydrophobic alkyl chains of the QAC salt interact with the phospholipid bilayer. This increases membrane permeability and induces the release of autolytic enzymes, resulting in bacterial cell lysis (adapted from [12,13]).

QACs are biocidal agents commonly used within domestic and industry environments (Figure 1). They are bactericidal across a range of microorganisms, including fungi, bacteria, parasites and lipophilic viruses [10]. Due to their aliphatic nature, QACs act as cationic surfactants; therefore, they destabilize the cell membranes and enzymes of target microorganisms, resulting in cell lysis [11,12,13]. Examples include benzalkonium chloride and cetylpyridinum chloride, both of which can target Gram-negative and Gram-positive bacteria such as *Escherichia coli* and *Staphylococcus aureus*, respectively [14]. The general structure represented as N + R^1^R^2^R^3^R^4^X− comprises a halide anion, commonly Cl^−^ or Br^−^, attached to a nitrogen cation [12]. 

**Figure 2:**
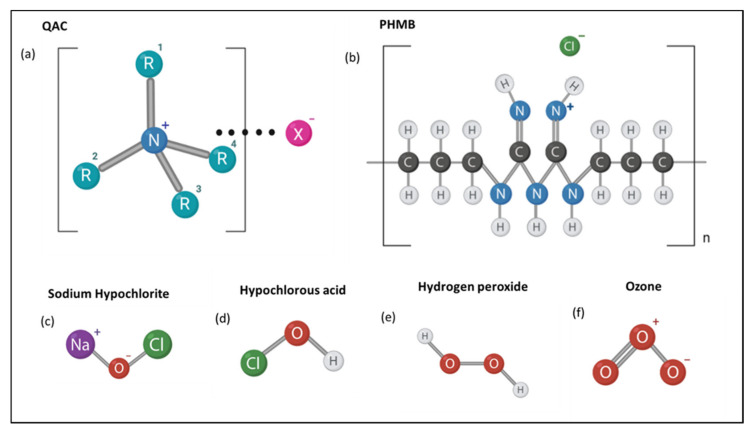
Molecular structure of common biocides in this review. The general structures of (**a**) QACs (Quaternary Ammonium Compounds), (**b**) polyhexamethylene biguanides (PHMB), (**c**) sodium hypochlorite, (**d**) hypochlorous acid, (**e**) hydrogen peroxide and (**f**) ozone are depicted.

Variations within the R group, such as the addition of akyl or aromatic groups, alter the QAC function (Figure 2a). For example, QACs with methyl groups from C_12_ to C_16_ elicit the highest biocidal activity, as do changes in the R groups [12]. Research is ongoing to understand the exact biocidal mechanism of QACs. Despite this, current understanding describes the electrostatic attraction of the QAC salt to the target cell bilayer and subsequent membrane disruption, leading to the release of autolytic enzymes which initiate cell lysis (Figure 1) [13]. QACs, such as benzalkonium chloride, act upon microbial membranes irrespective of their species. Therefore, they are also active against the collection of ESKAPE pathogens, including *Enterococcus faecium*, *Staphylococcus aureus*, *Klebsiella pneumoniae*, *Acinetobacter baumannii*, *Pseudomonas aeruginosa* and Enterobacter species, which demonstrate increased levels of antimicrobial resistance [14,15,16]. 

However, QAC biocides are not always effective for clinical use due to the formation of biofilms, such as those of *P. aeruginosa*, which have demonstrated increased resistance to QACs; thus, novel applications of QACs are being developed [16]. An example of this are the gemini QAC biocides, which contain two hydrophilic and hydrophobic ends as opposed to one, which have been developed to effectively induce biofilm bacterial cell lysis [17].

QACs have also been implemented for use as biocides within industry to decontaminate and prevent the spread of infections. Within the food industry, for example, benzyldimethyldodecylammonium chloride (BAC 12), benzyldimethyltetradecylammonium chloride (BAC 14) and benzyldimethylhexadecyl ammonium chloride (BAC 16) are used as surface decontaminants inside of milk transportation tanks used in dairy production. Such decontamination is imperative for public safety by preventing cross contamination and transmission of non-human pathogens. The suitability of QACS such as the aforementioned BAC 12–16 is due to their low toxicity levels, deeming it to be safe for the public especially under the EU regulation of 0.01 mg/kg QAC residue during food processing [1]. Unlike oxidizing biocides, such as those containing hydrogen peroxide, QACs do not produce free radicals; thus, they are not carcinogenic or genotoxic [1]. Hence they are useful as biocides within the home: cetylpyridinium chloride and dodecyl dimethyl benzyl ammonium chloride can be found within common cleaning fluids because they are active against a variety of bacteria at a low cost [7].

The efficacy of QACs at decontaminating surfaces is reliant upon factors including (i) biocide concentration, (ii) contact time of the biocide against the surface, (iii) the organic load, (iv) biocide formulation, (v) the surface temperature, (vi) the surface pH, (vii) whether a biofilm is present, and (viii) the type and number of microorganisms present on the surface to be decontaminated [18]. Dawson et al. [19] demonstrated how such factors may affect QAC efficacy when examining the Gram-positive bacterium *Clostridioides difficile*. The QAC biocides Newgenn^®^ (active agent Di-decyl dimethyl ammonium chloride) and Proceine-40^®^ (active agent alkyl-amino-alkyl glycines) were most effective against *Clostridioides difficile* spores and vegetative cells of Polymerase Chain Reaction (PCR) ribotype 027 (hypervirulent) strains as opposed to others, demonstrating that these biocides are strain-specific in activity. Conversely, the efficacy of Biocleanse^®^ (active agent benzalkonium chloride) was shown to be dependent upon both *C. difficile* strain PCR ribotype and biocide concentration; the clearance of ribotype 027 was most successful at a Biocleanse^®^ concentration of 5%, whereas the clearance of ribotype 017 was most successful at a concentration of 10%.

The consideration of how the biocide is applied to the contaminated surface is critical for appropriate disinfection. QAC formulations are commonly incorporated into wipes or sprays to be applied to the surface. In a study by Westgate et al. [20], the QAC formulation containing alkyl (C12–16) dimethylbenzylammonium-chloride presented greater activity dependent on the material of the wipe, although this may not affect the biocide efficacy [11]. The time taken to wipe a surface can also affect efficacy as demonstrated by Williams et al. [18], who established that although the QAC-formulated Clinell Universal Sanitizing Wipes had effective biocidal properties against surfaces loaded with Methicillin-resistant *Staphylococcus aureus* (MRSA) and Methicillin-sensitive *Staphylococcus aureus* (MSSA), these Gram-positive bacteria were able to survive on the wipes. Thus, secondary use of these wipes would negate their biocidal efficacy. It is clear that the application methods of QACs to surfaces to reduce bioburden, alongside the time of contact, are important for biocide efficacy and bacterial control.

## 3. Biguanides

The most common biguanide biocides include chlorhexidine digluconate (CHG) and polyhexamethylene biguanides (PHMB). Chlorhexidine is used across a variety of applications from hand hygiene and washing patients to antiseptic rinses for the oral cavity [21]. The primary concentration used for antisepsis is 0.02–4% *v/v* and for surface disinfection 0.5–0.4% *v/v* [1]. Its mechanism of action is via damaging the bacterial cytoplasmic membranes causing leakage of the bacterium’s cytoplasmic contents [22]. However, considerable evidence of bacterial resistance to CHG has emerged in recent years, ranging from changes in the bacterial cell membranes to withstand the effects of CHG, to the use of efflux pumps [3,23,24]. The use of CHG within fields such as dentistry has arguably allowed for selective pressure and CHX resistance to emerge in key oral bacteria, such as *Streptococcus sanguinis* and *Enterococcus faecalis* [25].

Polyhexamethylene biguanide has the structure shown in Figure 2b with varying end groups of guanide or cyanoguanide [26]. Bacteriostatic at low concentrations, PHMB is similar to QACs in that it is an amphipathic compound, cationic in nature and uses similar modes of activity to QACs. PHMBs are also bactericidal at higher concentrations [26]. The biocidal mechanism of PHMB involves adherence to lipids within the target cell membrane and subsequent non-specific disruption of components within the membrane. [26,27]. This broad antimicrobial specificity of PHMB has enabled it to be applied to the food, health and water hygiene industries for the sanitization of surfaces. It is regarded as safe to use within industry due to its low toxicity levels to humans. Unlike in prokaryotes, eukaryotic cells present greater compartmentalization and eject the biocide from the nucleus. Therefore, a greater Minimum Inhibitory Concentration (MIC) of PHMB is required for human eukaryotic cells than for the microorganisms’ prokaryotic cells; thus, human cells can withstand the concentrations of biocide required for decontamination [27,28,29,30].

PHMB serves multiple uses within the health industry and clinical settings in disinfecting wounds (commonly as a combination of 0.1% PHMB and 0.1% betaine), dressings and utensils; PHMB may also be used for the disinfection of biofilms on medical equipment or surfaces. Machuca et al. [27] demonstrated that PHMB-betaine solution was active against Gram-negative and Gram-positive bacteria, including biofilms of *Klebsiella pneumoniae* ST-716, *Acinetobacter baumannii* and *S. aureus*, all of which are of clinical concern due to rising antimicrobial resistance. This broad spectrum of activity both Gram-positive and Gram-negative bacteria has led to the use of PHMB against Mycobacterium species, including *Mycobacterium smegmatis* at an MIC of 5 mg/L for example [29]. Ongoing research aims to determine the suitability of PHMB as an antiseptic in wound dressings; Hübner et al. [30] found that the presence of organic matter such as cartilage may affect the efficacy of PHMB against *E. coli* and *S. aureus*. Despite this, PHMB-containing disinfectants can prevent secondary bacterial infections and do not prevent wound re-epithelialization [30,31,32].

The context in which PHMB is applied also impacts its biocidal efficacy. In a study by Ng et al. [28] PHMB was incorporated into different nanofiber membranes used in water filtration: electrospun polyacrylonitrile nanofiber membranes were either directly coupled to PHMB molecules (P-COOH-PHMB membranes) or were modified by chitosan before PHMB incorporation (P-COOH-CS-PHMB membranes). The membranes were then placed over agar streaked with *E. coli*. Both membranes demonstrated >99.99% activity against *E. coli* at a PHMB concentration of ~1.75 mol/g membrane. However, following repeated exposure, P-COOH-CS-PHMB was less effective than P-COOH-PHMB due to poorer stability. The length of *E. coli* exposure also affected efficacy. For example, the activity P-COOH-PHMB and P-COOH-CS-PHMB increased by 43.14% and 17.37% when the contact time was increased from 5 to 10 min. Nevertheless, P-COOH-CS-PHMB was the most effective at both exposure times and was 29.35% more effective after 5 min of exposure compared to P-COOH-PHMB [29]. Indeed, another study by Renzoni et al. (2017) [32] found that PHMB was effective at decolonization of chlorhexidine-resistant strains of *S. aureus* strains at low PHMB concentrations, demonstrating the utility of PHMB as an antiseptic.

## 4. Chlorine-Releasing Agents

Chlorine-releasing agents (CRAs) are oxidizing agents that include sodium hypochlorite, hypochlorous acid and sodium dichloroisocyanurate. Sodium hypochlorite (NaOCl) is a strong electrolyzed water solution produced by the electrolysis of sodium chloride and contains 5–12% of available chlorine [33,34] (Figure 2c). When this basic solution is added to water, the hypochlorite partly dissociates into hypochlorite ions (^−^OCl) while the rest remains as hypochlorous acid (HOCl). Both OCl and HOCl are strong oxidizing agents; for example, they can oxidize the sulfhydryl groups of enzymes, which leads to impaired DNA and protein synthesis [35]. They also react with amino acids, such as methionine and cysteine, peptides, and DNA itself. Oxidative damage to membrane proteins may alter membrane permeability and transport capacity. This can allow microbial entry of the oxidative species generated by HOCl, which can then damage organelles. For example, the lethality of sodium hypochlorite to *E. coli* is due to the denaturation of sulfhydryl enzymes and antioxidants such as glutathione. This impairs cellular function, leading to cell death. This biocidal mechanism applies to a variety of CRAs, including N-chloramines [35,36].

CRAs are also commonly found in many household disinfectants. Sodium hypochlorite, for example, is commonly used within household bleach when diluted and is fit for this purpose as it has a shelf life of at least one month at average household temperatures and is the most stable CRA with a pH of 9–11. Its recommended concentration in Europe is 0.5% (5000 μg/mL) [36]. Novel disinfectant sprays containing electrolyzed water with chlorine are significantly less stable; however, they have been shown to be effective at decontaminating kitchen surfaces from *S. aureus* and *E. coli*. Sodium hypochlorite solution is also used frequently to decontaminate healthcare facilities soiled with pathogenic bacterial spores of *Clostridioides difficile* (formally known as *Clostridium difficile*) [37,38,39].

Sodium dichloroisocyanurate is only stable as a solid, not as a solution; these unstable CRAs are, thus, more likely to be found in industry than in the home [40]. For example, CRAs are used within hospitals to prevent hospital-acquired infections and are used at sporicidal concentrations of 1000 ppm, 5000 ppm and 10,000 ppm of active chlorine, usually in tablet form [38]. For example, guidelines recommend 1000 ppm or 5000 ppm active chlorine for 10 min to be used for disinfection of surfaces laden with *C. difficile* spores; however, recent data suggest that *C. difficile* spores (ribotypes 027, 012) can survive exposure to Sodium dichloroisocyanurate at 1000 ppm, and thus, the utility of CRAs at this concentration has been called into question [40,41]. In response to spores of *C. difficile* (ribotypes 012, 017 and 027), it has been found that the CRAs are only effective at high concentrations. Dawson et al. (2011) [19] demonstrated that Actichlor^®^ and Haztabs^®^ (both contain the active agent sodium dichloroisocyanurate), at a concentration of 5000 ppm, were able to eradicate spores of all ribotypes below detectable levels, whereas at a concentration of 1000 ppm, the spores of all ribotypes survived. Other sporicidal CRAs include chlorine dioxide and hypochlorite, which degrade the cortex peptidoglycan and spore coat of dormant spores causing them to lyse upon germination [38,39].

Hypochlorous acid (HOCl) is inexpensive, generally toxic and can be used within mouthwashes, sanitizers, clinical disinfection at 1000 ppm, podiatry and as a part of wound care [42] (Figure 2d). Interestingly, it is also generated by the human immune system as part of the initial innate immunity defense against infectious agents [43]. While there is limited evidence regarding bacterial resistance to HOCl, it has been noted that HOCl exposure can cause the formation of biofilms in Gram-negative bacteria through the over production of extracellular polymeric substances (EPS) [44]. There has, however, been no reported cases of bacterial resistance to hypochlorous acid to date. Another attribute of CRA use in hospitals is their efficacy against common antibiotic resistant strains: 0.01% and 0.1% sodium hypochlorite can kill MRSA- and MSSA-contaminated surfaces.

Generally, CRA biocide activity presents greater efficacy on non-porous, smoother surfaces such as stainless steel 304 and nitrile compared to porous surfaces such as wood or rubber [45]. Another major factor that decreases the efficacy of CRAs is the presence of organic materials. Therefore, the cleaning and removal of organic matter before disinfection is recommended [40]. However, in cases where this is not possible, specific guidelines may be followed. For example, The Australian Pesticides and Veterinary Medicines Authority suggest that in the presence of organic material, a 1% concentration of sodium hypochlorite is required for the acceptable decontamination of *Mycobacterium bovis* [44]. In the absence of organic material, only 0.04% sodium hypochlorite is required, further demonstrating the significance of organic material in CRA surface decontamination [44]. Moreover, due to an increase in chlorine availability, sodium dichloroisocyanurate can be more tolerant, and thus, more effective in the presence of organic material.

Chlorine content, pH level and redox potential can further affect CRA efficacy. Hypochlorous acid presents high oxidizing activity, and thus, a high redox potential, enabling a greater production of reactive oxygen species. As demonstrated by Severing et al. [45], CRA biocide products such as Microdacyn60^®^ and Veriforte™ contain low total chlorine quantities of 80 ppm and 93 ppm. Contrastingly, the products containing no hypochlorous acid but instead just sodium hypochlorite, such as KerraSol™ and Lavanox^®^, present high total chlorine quantities of 690 ppm and 670 ppm. These products also read at a higher pH compared to Microdacyn60^®^ and Veriforte™. After exposure to *S. aureus* and *P. aeruginosa*, KerraSol™ and Lavanox^®^ were the more effective disinfectants [44]. As a result, Severing et al. [45] indicated that biocide pH and total chlorine availability present the greatest influence over biocidal efficacy compared to redox potential and oxidizing activity.

## 5. Hydrogen Peroxide

Hydrogen peroxide is another powerful oxidizing agent [46,47] (Figure 2e). Radicals produced by reactions with hydrogen peroxide act on a range of microbial target sites, both extracellular and intracellular. Oxidation by hydroxyl radicals, for example, of polyunsaturated acids within membrane phospholipids results in cell lysis and subsequent oxidation of the released cellular components. Due to their low molecular weight, hydrogen peroxide molecules can traverse through microbial cell walls and membranes to act intracellularly without having first induced cell lysis. The hydroxyl radicals then oxidize thiol groups of intracellular proteins, enzyme, lipids and nucleosides within DNA [47,48,49]. Although the main biocidal mechanisms elucidated include radical induced membrane damage, intracellular protein damage and DNA damage, more research is required into which mechanism is the leading cause of hydrogen peroxide-induced cell death when applied as a biocide [49].

Hydrogen peroxide is typically unstable, and thus, difficult to store; hence, it presents many advantages for use in decontamination. For example, it only degrades into water and hydrogen, making it an environmentally friendly choice as a disinfectant within industries such as the food industry; a common commercial disinfectant used is Sanosil-25, which contains 0.24% hydrogen peroxide. It is also non-toxic, and thus, is safe to use as a disinfectant for medical equipment and surfaces; a solution of 3–6% hydrogen peroxide in water is commonly used [49,50]. Furthermore, hydrogen peroxide is active against a variety of microorganisms including bacteria, yeasts and viruses [50]. Not only can hydrogen peroxide be applied to surfaces in aqueous form, but also in vaporized form by a process called fumigation. The cytotoxic mechanism differs depending on the liquid/vapor state and this affects the biocidal activity. For example, unlike aqueous hydrogen peroxide, in the vaporized form, it is unable to oxidize amino acids, yet this form is more efficient at protein oxidation [48]. Hydrogen peroxide vapor can be beneficial, as it has been shown to be effective at decontaminating clinical surfaces and equipment within hospital rooms infected with MRSA and *C. difficile*. However, decontamination with this method can be impractical and the application of liquid hydrogen peroxide is still commonplace [50].

Kenters et al. [51] demonstrated the impact of different application methods on the biocidal efficacy of hydrogen peroxide products. Each medium contained 1.5% active hydrogen peroxide and was either sprayed or wiped onto ceramic tiles infected with *C. difficile* spores of Ribotypes 027, 014 and 010. Both the sprays and wipes reduced colony forming unit (CFU) counts for all ribotypes; for example, a wipe containing hydrogen peroxide at 1.5% concentration resulted in a 5 log_10_ CFU reduction. However, generally lower CFU reductions were found for the clinically important ribotypes 027 and 014 than the non-toxic 010 ribotype, although this is variable depending on the level of organic contamination [51]. Moreover, a significant difference in *C. difficile* decontamination was found depending on how the product was applied to the surface, with wipes resulting in greater CFU reductions than the sprays: wipes containing accelerated hydrogen peroxide produced log_10_ CFU reduction of 5.29 compared to the spray, also containing accelerated hydrogen peroxide, which produced log_10_ CFU reduction of 4.08 [51]. Thus, the importance of the application method and microorganism strains to be disinfected is highlighted.

It is also necessary to consider the material of the wipe, as this may impact the quantity of the product adsorbed onto the wipe. Westgate et al. [20] found hydrogen peroxide-containing microfiber wipes and non-woven wipes to be more effective against *S. aureus* and *P. aeruginosa* than cotton wipes. Biocide products commonly contain a mixture of components to enhance efficacy. A study by Ríos-Castillo et al. [52] recommended a combination of 3.0% hydrogen peroxide alongside 1.0% monophenyl glycol, 0.3% acetophosphonic acid and 3.5% lactic acid formulated with cationic polymer for the disinfection of *S. aureus* and *P. aeruginosa*. This formula due to a reduced pH is more effective at reducing bacterial growth than hydrogen peroxide alone. It also has a broad specificity against both Gram-positive and Gram-negative bacteria and may be beneficial for use in humid environments [52]. Furthermore, hydrogen peroxide demonstrated enhanced activity against *S. aureus* and *P. aeruginosa* biofilms when delivered in micelles. At a concentration of 1.7% with 5 min exposure, the hydrogen peroxide resulted in a 1.5 log_10_ CFU reduction compared to > 8 log_10_ CFU reduction when encapsulated within micelles [52].

Whether the hydrogen peroxide is applied in liquid form, vapor form or even a foam affects its efficacy. A study by Le Toquin et al. [53] found hydrogen peroxide added to foam to be more effective at higher temperatures at inactivating *Bacillus thuringiensis* spores compared to its liquid counterpart. However, the temperature sensitivity of the foam affects the contact time required; when applied to a vertical surface, the biocide was effective after 25 min at 30 °C but not at 4 °C, for which 2 h and 30 min was calculated as required for effective disinfection [53]. Due to the ability of vapor and foam-based biocides to decontaminate difficult to reach surfaces, they may be more beneficial for the decontamination of whole rooms, for example patient rooms in hospitals.

## 6. Ozone

Similar to hydrogen peroxide, ozone is a strong oxidizing agent active against a range of both Gram-positive and Gram-negative bacteria, viruses, fungi and protozoa [54] (Figure 2f). Ozone induces bacterial cell lysis via the oxidation of membrane phospholipids and lipoproteins, such as within the Gram-positive membrane of *Listeria monocytogenes* [54]. Because ozone can dissolve within solution or be applied in gaseous form, it can be widely used in industry, especially to treat wastewater [55].

Ozone gas presents many advantageous: it is easy to produce, has a 20-min half-life and can disinfect places which are difficult to reach using conventional solution-based biocides. However, ozone can be toxic at high concentrations; thus, the room to be decontaminated must be quarantined [56]. Additionally, the presence of organic matter may affect decontamination depending on whether ozone is gaseous or aqueous. In the presence of serum, the efficacy of ozonated water when applied to *L. monocytogenes* was reduced [56,57]. Ozone gas may be used for the disinfection of hospital rooms or transport vehicles, whereas dissolved ozone may be used in water treatment and food disinfection (Table 1) [58].

## 7. Emerging Biocide Resistance and Impacts on AMR

In this review, we have outlined uses of common biocides, their activity and evidence of emerging bacterial resistance (Table 1). There are still limitations to our current breadth of knowledge regarding biocide resistance and antimicrobial resistance. Biocides are used significantly across healthcare and industry to control microbial contamination, especially now within the antibiotic era; however, their overuse, especially at inappropriate concentrations, could contribute to an increase in bacterial resistance to antimicrobials [1,41]. Due to the limited studies in the area, there is a dearth of knowledge regarding selective pressure and bacterial biocide resistance; however, in contrast, it is well-known that intensive use and misuse of antibiotics causes antibiotic resistance [1]. Indeed, studies have sought to examine whether biocide resistance and antibiotic resistance are intrinsically interlinked, and while it is clear that selective pressure may play a key role in the emergence of high and low level of biocide resistance in certain bacteria, more studies must be conducted to understand the full impacts of co-resistance [5,6,7,8,9].

A good example of the above is a study conducted by Wesgate et al. [20], where clinical antibiotic resistances were assessed against common biocides. The study found that the bacterial strains tested did not maintain stable clinical antibiotic resistance and there was limited understanding of the mechanisms involved in co-resistance of biocides and antibiotic resistance. This is not to suggest that potential mechanisms of resistance have not been identified, such as efflux pumps, horizontal gene transfer and mutations; rather, these mechanisms have not yet been widely studied across a range of representative clinical pathogens [7,20,58]. Additionally, the effects of pH, temperature and presence of organic bioburden have not been extensively studied. Thus, further studies, implementation and design of interventions and surveillance programs are strongly encouraged to ascertain what the impacts of overuse of biocides may have on antimicrobial resistance as a whole.

## 8. Conclusions

Biocides are being increasingly used as choice agents for chemical antimicrobial disinfection across healthcare, home and industrial environments. Their inappropriate use could lead to selective pressure, resulting in the emergence of resistance alongside the general antimicrobial resistance (AMR) currently happening at a global scale. More research is needed to understand the true effects of this increased use in practice and rationalization and appropriate use of biocides for disinfection of surfaces from microorganisms is encouraged.

## Figures and Tables

**Table 1:** Mode of action, advantages and disadvantages of biocides.

Biocide	Mode of Action	Advantages	Disadvantages
Quaternary Ammonium Compounds	Cationic action destabilizes cell membrane resulting in cell lysis [11,12,13,14].	Does not produce free radicals; therefore, they are not carcinogenic or genotoxic [11,12]. Generally inexpensive to use [1].	Less effective against biofilms [16]. Efficacy can be strain specific [19]. Efficacy may vary with temperature [17,20].
Polyhexamethylene Biguanides	Adherence to lipids within cell membranes leading to non-specific cell membrane disruption, allowing cellular entry of PHMB [25,26].	Broad antimicrobial specificity [24]. Low toxicity [25,26,27]. Water soluble, thermostable and pH stable [26]. Presents activity against certain biofilms including that of antimicrobial resistant strains [27].	Efficacy is temperature sensitive [28]. Efficacy may be altered by presence of organic matter [29,31].
NaOCl	Oxidative damage to cell membrane, as well as intracellular proteins and amino acids. Membrane damage leads to entry of NaOCl to damage organelles [33,35].	Suitable for household use due to appropriate shelf life and stability at average household temperatures [34,35]. Safe for human hygiene [35].	Efficacy may be altered by presence of organic matter [38]. Efficacy may be altered depending on contaminated surface material [41,47,48].
ClO_2_ (chlorine dioxide gas)	Oxidative damage to cell membrane, as well as intracellular proteins and amino acids. Membrane damage leads to entry of ClO_2_ to damage organelles [33].	Safe for human hygiene. Not cytotoxic. Can be active against biofilms. Oxidative mechanism is greatly specific thus less product is required. [58]	Gas generation is expensive [58]
Hypochlorous acid (HClO)	Oxidative damage to cell membrane, as well as intracellular proteins and amino acids. Membrane damage leads to entry of HClO to damage organelles [33,46].	Generally inexpensive and non-toxic [33]. Safe for human hygiene [46]. Can be effective against enveloped viruses [58].	Reduced oxidative specificity means more product is required [58].
Peroxides (H_2_O_2_)	Hydroxyl radicals cause oxidative damage to cell membrane components as well as intracellular molecules [48,49].	Only degrades into water and hydrogen—environmentally friendly [48]. Broad antimicrobial specificity [55]. Can be applied in aqueous or vaporized form [54]. Vaporized form enables disinfection of ‘hard to reach’ places [53,54].	Typically unstable therefore difficult to store [54]. Presents strain specificity [49]. Efficacy varies with application method [48].
Ozone (gas)	Induces cell lysis via membrane oxidation [56].	Broad antimicrobial specificity [55]. Easy to produce with a 20-min half -life [56]. Enables easier disinfection of ‘hard to reach’ places [56].	Toxic at high concentrations [55]. Efficacy may vary in the presence of organic matter depending on whether the ozone is in gaseous or aqueous form [55,57].
